# Beyond surface dose: subcutaneous dose escalation underlies TomoTherapy’s skin toxicity in breast radiotherapy

**DOI:** 10.3389/fonc.2026.1760716

**Published:** 2026-01-21

**Authors:** Jiao Xue, Wenjun Zhao, Chenchen Wu

**Affiliations:** 1 Department of Radiation Oncology, The First Affiliated Hospital of Soochow University, Suzhou, China; 2 State Key Laboratory of Radiation Medicine and Protection, School for Radiological and Interdisciplinary Sciences (RAD-X), Collaborative Innovation Center of Radiation Medicine of Jiangsu Higher Education Institutions, Soochow University, Suzhou, China

**Keywords:** acute skin toxicity, breast cancer radiotherapy setup, helical tomotherapy (TOMO), P-PAST, volumetric modulated arc therapy (VMAT)

## Abstract

**Purpose:**

To elucidate the dosimetric mechanisms underlying the increased incidence of acute radiation dermatitis associated with Helical TomoTherapy (TOMO) compared to Volumetric Modulated Arc Therapy (VMAT) in whole-breast irradiation.

**Methods and materials:**

A retrospective analysis included 40 patients with right-sided breast cancer receiving hypofractionated whole-breast irradiation (40 Gy/15 fractions), evenly matched between VMAT (Elekta Synergy) and helical TomoTherapy (TOMO) groups. Skin dose was evaluated using a multi-parametric approach: 1) Dose Surface Maps (DSM); 2) a novel 5-mm thick PTV-projected adjacent superficial tissue (P-PAST) structure; and 3) equivalent dose in 2-Gy fractions (EQD_2_, α/β=10). Clinical skin toxicity was graded per RTOG criteria.

**Results:**

Clinically significant (Grade 2-4) acute skin toxicity was substantially more frequent with TOMO (70%) than with VMAT (30%, p=0.026). DSM showed TOMO produced a more confined high-dose region on the skin surface compared to VMAT. However, TOMO delivered significantly higher physical and biologically effective doses to the subcutaneous P-PAST region: mean EQD_2_ was 44.47 ± 1.33 Gy vs. 42.12 ± 0.76 Gy for VMAT (p<0.001). D2cc and maximum dose EQD_2_ were also elevated in the TOMO group.

**Conclusions:**

The heightened skin toxicity with TOMO is not predicted by conventional skin surface dose metrics but is explained by a significant dose escalation within the subcutaneous P-PAST region. Plan evaluation for TOMO should incorporate volumetric dose assessment of the dermal compartment to mitigate toxicity.

## Introduction

The global incidence of breast cancer has been rising annually, and with an estimated 2.3 million new cases in 2020, it has surpassed lung cancer to become the most frequently diagnosed cancer worldwide (1). Since landmark trials in the 1980s demonstrated its efficacy, the combination of breast-conserving surgery and radiotherapy has served as a fundamental treatment option for early-stage breast cancer for over thirty years (2–6). Advanced techniques such as Intensity-Modulated Radiotherapy (IMRT), Volumetric Modulated Arc Therapy (VMAT), and Helical Tomotherapy (TOMO) have become mainstream modalities for post-operative radiotherapy in breast cancer, with each exhibiting distinct dosimetric characteristics (7–10).

Among these, TOMO is frequently recognized for its superior capabilities in achieving highly homogeneous target coverage and reducing radiation dose to deep-seated organs at risk, such as the lungs and heart (11–13). Paradoxically, this technical prowess for protecting deep-seated organs appears to incur a cost at the skin surface. Emerging clinical observations and studies suggest that, compared to VMAT, TOMO is associated with a higher incidence and severity of acute radiation dermatitis in breast cancer patients (14–16). The underlying dosimetric mechanisms for this apparent trade-off, where better deep tissue sparing correlates with worse skin toxicity, remain poorly understood and constitute a significant clinical puzzle.

This study aims to systematically compare the skin dose of VMAT and TOMO by employing the Dose Surface Mapping (DSM) approach, as described by Patrick et al. (17), to visualize the global distribution pattern, combined with a novel analysis of the PTV-projected adjacent superficial tissue (P-PAST) to quantify the dose intensity in the skin region most relevant for toxicity. This approach is designed to explicitly link TOMO’s unique delivery geometry to the observed increase in clinical skin toxicity.

## Methods and materials

### Patient cohort

A retrospective analysis was conducted on 40 breast cancer patients with right-sided disease who underwent breast-conserving surgery, treated with radiation therapy between January 2022 and December 2023. All patients had early-stage right breast cancer and were treated with a hypofractionated regimen of 40 Gy in 15 fractions. The cohort was divided into two matched groups: 20 patients were treated with Volumetric Modulated Arc Therapy (VMAT) on an Elekta Synergy linear accelerator, and 20 patients were treated on an Accuray TomoTherapy helical radiotherapy system.

Patient selection was based on stringent matching criteria to ensure comparability between groups. The inclusion criteria comprised (1): histologically confirmed invasive breast carcinoma or ductal carcinoma *in situ* located in the right breast (2), status post breast-conserving surgery with clear margins (3), receipt of hypofractionated whole-breast irradiation (40 Gy in 15 fractions) to the right breast, and (4) availability of complete dosimetric data and clinical follow-up records. The two groups were well-balanced in terms of baseline and dosimetrically relevant parameters, including patient age and planning target volume (PTV), as detailed in the Results section.

To ensure the comparability of the two treatment groups and to account for anatomical factors known to influence superficial dose, key patient-specific metrics were evaluated. The body mass index (BMI) was calculated for all patients from their recorded height and weight. Additionally, breast separation was measured as an indicator of chest wall geometry, which can affect dose distribution in the skin and subcutaneous tissues. The measurement of breast separation was performed according to a standard methodology used in breast radiotherapy dosimetric studies (18). This metric represents the transverse breast width. All linear measurements were conducted within the 3D Slicer software platform (version 5.6.2) using the Create Line tool in the Markups module.

### Delineation of targets and normal tissues

The clinical target volume (CTV) was contoured by the attending radiation oncologist according to institutional guidelines. The planning target volume (PTV) was generated by applying a 5 mm isotropic margin to the CTV, followed by a 3 mm crop from the body surface to account for the dose build-up effect. Normal tissue structures were generated automatically using the United Imaging Healthcare (UIH) Radiotherapy Contouring Software (uPWS, complete version R001.3.0.371767). The initial External contour was created using the ‘Other’ module, while other normal tissues (e.g., lungs, heart, contralateral breast) were generated from the ‘Chest’ module within the intelligent segmentation interface. All auto-generated contours were subsequently reviewed and manually adjusted as necessary by qualified radiation oncologists.

### Dosimetric evaluation of organs at risk

To quantify the dose to deep-seated organs and evaluate the dosimetric trade-off, dose-volume histogram (DVH) parameters for key OARs were compared between techniques. Based on the contours generated and reviewed as described in the Delineation section, the following parameters were extracted:

*Ipsilateral Lung:* The volume receiving at least 5 Gy (V5), the volume receiving at least 20 Gy (V20), and the mean dose (Dmean).

*Heart:* The volume receiving at least 5 Gy (V5), the volume receiving at least 25 Gy (V25), and the mean dose (Dmean).

### Treatment planning system and delivery technique

Treatment planning and delivery techniques were platform-specific.

For the Synergy/VMAT group, all plans were optimized and calculated using the Monaco treatment planning system (version 5.51.11). To achieve optimal dose conformity and efficient delivery, a four-partial-arc technique was employed. The specific arc configuration was as follows: two counter-clockwise arcs (gantry rotation from 70° to 350° and from 285° to 190°) and two clockwise arcs (gantry rotation from 190° to 285° and from 350° to 70°). The collimator angle was set to 0°for all arcs. Critically, to ensure robust dose coverage of the breast tissue up to the skin surface despite breathing motion and setup uncertainties, the Monaco system’s “autoflash” feature was enabled during optimization for all VMAT plans. This feature dynamically extends the multileaf collimator (MLC) positions beyond the body contour as needed during the arc rotation, creating a dynamic “flash” margin that guarantees sufficient superficial dose, thereby accurately representing the skin-sparing compromise inherent in clinical treatment.

For the TomoTherapy group, treatments were delivered using the TomoTherapy system (software version 1.1.1.1). Plans were generated employing a dynamic jaw mode and a fixed field width of 5.0 cm. The pitch factor was typically set at 0.287, and the modulation factor was constrained between 2.0 and 2.5 for all plans. The TomoTherapy planning system does not employ an equivalent “flash” feature; its helical delivery inherently treats the surface through continuous rotation from all angles.

### Multi-parametric skin and subcutaneous dose evaluation methods

To systematically evaluate the dose distribution in the skin and underlying tissues, this study employed the following three complementary analytical approaches:

(1) Skin Surface Maximum Dose Analysis

To visually compare the dose distribution patterns on the skin surface between the two techniques (VMAT and TOMO), a pair of representative patients with the most closely matched PTV volumes (Synergy: 1040.80 cm³; TOMO: 1050.18 cm³) were first selected for qualitative analysis.

A Dose Surface Map (DSM) was generated using a custom Python-based processing pipeline. The process involved: extraction of the external body surface from the planning CT dataset, generation of a surface mesh using a high-resolution voxel grid (1.5×1.5×2.5 mm³), projection of the 3D dose distribution onto the surface mesh using ray-tracing algorithms, and visualization using a standardized color scale (inferno colormap) to ensure consistent comparison across patients. The maximum dose (Dmax) extracted from this DSM was compared between the two groups.

(2) PTV-Projected Adjacent Superficial Tissue (P-PAST) Analysis

To evaluate the dose to the most superficial part of the target, a structure termed the PTV-projected adjacent superficial tissue (P-PAST) was defined. This was achieved by first shrinking the External contour inward by 5 mm to create a skin layer. This skin layer was then Boolean-intersected with the final PTV to generate the P-PAST, which constitutes a sub-region of the PTV directly beneath the skin surface. The 5 mm depth was selected based on previous literature (19, 20), as it encompasses the biologically relevant skin layers (epidermis and dermis) responsible for acute radiation skin reactions. The mean dose (Dmean), D2cc, and maximum dose (Dmax) within the P-PAST were compared between the two groups. All dose-volume parameters were extracted and analyzed using the open-source platform 3D Slicer (version 5.6.2) and were verified with independent calculations.

### Dosimetric parameters and biologically effective dose analysis

To compare the biological effectiveness of the differential dose distributions within the P-PAST region between the two techniques, the equivalent dose in 2-Gy fractions (EQD_2_) was calculated for the P-PAST parameters (Dmean, D2cc, and Dmax). This conversion accounts for the differences in dose per fraction, providing a unified scale to assess the potential for causing early radiation skin toxicity.

The EQD_2_ was calculated using the Linear-Quadratic model:


$${\text{EQD}}_{2}=\text{D }\times \;\left[\left(d+\;\frac{\alpha }{\beta }\right)/\left(2+\frac{\alpha }{\beta }\right)\right]$$

where D is the total physical dose to the P-PAST structure, d is the dose per fraction (d=D/15), and the α/β ratio was set to 10 Gy. This value is well-established for modeling the response of early-responding tissues, including the epidermis and dermis responsible for acute radiation dermatitis (21, 22).

The resulting EQD_2_ values for the mean dose, D2cc, and maximum dose within the P-PAST were subsequently compared between the Synergy/VMAT and TomoTherapy groups.

### Plan quality evaluation metrics

To ensure a fair dosimetric comparison and to rule out the possibility that observed skin dose differences were attributable to compromised target coverage, the quality of all treatment plans was evaluated using standardized metrics for the planning target volume (PTV).

*Conformity Index (CI):* The CI was calculated to assess how closely the prescribed high-dose volume conformed to the shape of the PTV, using the following widely adopted formula (23):


$$\text{CI}=\frac{{\left(TV_{PIV}\right)}^{2}}{\left(TV\;\times \;PIV\right)}$$

where TV_PIV_ is the volume of the PTV covered by the prescription isodose (i.e., receiving ≥ 95% of 40 Gy), TV is the volume of the PTV, and PIV is the Prescription Isodose Volume (i.e., the total volume receiving ≥ 95% of 40 Gy).

*Homogeneity Index (HI):* The HI was calculated based on the dose-volume metrics recommended in the ICRU Report 83 (24) using the following formula:


$$\text{HI}=\frac{D_{2\%}-D_{98\%}}{D_{50\%}}$$

where D_2%_, D_98%_, and D_50%_ represent the dose received by 2%, 98%, and 50% of the PTV volume, respectively. A lower HI value indicates a more homogeneous dose distribution within the target.

### Clinical skin toxicity assessment

To correlate the observed dosimetric differences with clinical outcomes, acute skin toxicity was assessed using the Radiation Therapy Oncology Group (RTOG) acute radiation morbidity scoring criteria. Patients were evaluated weekly during radiotherapy and every 2 weeks until 3 months after treatment completion. The highest toxicity grade observed during this acute phase (from treatment initiation to 3 months post-radiotherapy) was recorded as the primary endpoint for skin toxicity analysis. All assessments were performed independently by two radiation oncologists with over 5 years of experience in breast radiotherapy, who were blinded to the treatment technique. Any discrepancies in toxicity grading were resolved by consensus.

### Statistical analysis

All statistical analyses were performed using GraphPad Prism software (version 10.1.2). Continuous dosimetric parameters, patient age, and PTV volume are presented as mean ± standard deviation. Comparisons of these continuous variables between the Synergy and TOMO groups were performed using independent samples t-tests with Welch’s correction for potential unequal variances. A p-value of less than 0.05 was considered statistically significant.

## Results

### Patient characteristics

Baseline patient and treatment characteristics are summarized in **Table 1**. There was no statistically significant difference in patient age between the VMAT group (54.3 ± 14.3 years) and the TOMO group (59.4 ± 12.1 years; p = 0.236). The planning target volume (PTV) was also closely matched between the groups, with no significant difference in mean volume (VMAT: 721.7 ± 334.0 cm³ vs. TOMO: 667.5 ± 270.6 cm³; p = 0.577). Critically, key anatomical parameters known to influence superficial dose were also comparable. Body mass index (BMI) did not differ significantly between the VMAT and TOMO groups (23.7 ± 2.6 kg/m² vs. 24.5 ± 3.4 kg/m², p = 0.368). Similarly, the breast separation, a measure of chest wall width, was well-matched (18.0 ± 2.5 cm vs. 18.6 ± 3.0 cm, p = 0.510).

**Table 1:** Patient characteristics.

Characteristic	VMAT group (n=20)	TOMO group (n=20)	p-value
Age (years), mean ± SD	54.3 ± 14.3	59.4 ± 12.1	0.236
PTV volume (cm³), mean ± SD	721.7 ± 334.0	667.5 ± 270.6	0.577
BMI (kg/m²), mean ± SD	23.7 ± 2.6	24.5 ± 3.4	0.368
Breast Separation (cm), mean ± SD	18.0 ± 2.5	18.6 ± 3.0	0.510

### Plan quality comparison: target coverage and homogeneity

To ensure the dosimetric comparison was unbiased and that differences in skin dose were not attributable to compromises in target coverage, the plan quality for both techniques was rigorously evaluated. The Conformity Index (CI) and Homogeneity Index (HI) for the PTV were calculated and compared between the VMAT and TOMO groups (**Table 2**). The CI was significantly higher for VMAT plans (0.828 ± 0.046) compared to TOMO plans (0.780 ± 0.056; p = 0.009), indicating superior target conformity with VMAT. In contrast, there was no significant difference in the HI between the two groups (VMAT: 0.108 ± 0.042 vs. TOMO: 0.099 ± 0.026; p = 0.403), demonstrating comparable dose homogeneity within the PTV. These results confirm that the VMAT plans did not achieve lower skin doses at the expense of inferior target coverage or modulation; rather, they maintained excellent, if not superior, plan quality metrics.

**Table 2:** Comparison of plan quality metrics between VMAT and TOMO groups.

Metric	VMAT group (n=20)	TOMO group (n=20)	p-value
CI, mean ± SD	0.828 ± 0.046	0.780 ± 0.056	0.009
HI, mean ± SD	0.108 ± 0.042	0.099 ± 0.026	0.403

### Dosimetric comparison of organs at risk

To quantitatively evaluate the trade-off between superficial and deep tissue dose, the dose to critical OARs was compared between the VMAT and TOMO groups (**Table 3**).

**Table 3:** Comparison of organ at risk dosimetry between VMAT and TOMO groups.

Organ at risk	Parameter	VMAT group (n=20)	TOMO group (n=20)	p-value
Ipsilateral Lung	V5 (%)	36.8 ± 7.6	33.6 ± 9.1	0.287
	V20 (%)	17.0 ± 4.7	11.5 ± 4.2	0.001
	Mean Dose (Gy)	8.79 ± 1.75	7.32 ± 1.77	0.020
Heart	V5 (%)	5.6 ± 4.8	4.6 ± 5.5	0.684
	V25 (%)	0.1 ± 0.4	0.0 ± 0.0	0.339
	Mean Dose (Gy)	2.31 ± 0.64	1.67 ± 0.67	0.042

*For the ipsilateral lung:* The TOMO group demonstrated statistically significant reductions in the mean lung dose (7.32 ± 1.77 Gy vs. 8.79 ± 1.75 Gy, p = 0.020) and the volume receiving 20 Gy (V20: 11.5 ± 4.2% vs. 17.0 ± 4.7%, p = 0.001). The volume receiving 5 Gy (V5) was comparable between the two groups (33.6 ± 9.1% vs. 36.8 ± 7.6%, p = 0.287).

*For the heart:* In patients with relevant cardiac exposure, the TOMO group also showed a significantly lower mean heart dose (1.67 ± 0.67 Gy vs. 2.31 ± 0.64 Gy, p = 0.042). The low-dose parameters, V5 and V25, were not significantly different between the groups.

These results confirm that TOMO plans achieved superior sparing of deep-seated OARs in this cohort, thereby empirically establishing the specific dosimetric trade-off: enhanced protection of deep organs at the cost of increased dose to the subcutaneous dermal compartment.

### Clinical skin toxicity

The assessment of acute skin toxicity revealed a marked difference in toxicity profiles between the two treatment techniques. As detailed in **Table 4**, the distribution of RTOG grades was significantly skewed toward higher severity in the TomoTherapy group.

**Table 4:** Comparison of acute skin toxicity (RTOG Grading Criteria) between Synergy/VMAT and TomoTherapy Groups.

RTOG acute skin toxicity grade	VMAT group (n=20)	TOMO group (n=20)
Grade 0	0	0
Grade 1	14	6
Grade 2	4	9
Grade 3	2	4
Grade 4	0	1
Clinically Significant Toxicity (Grade 2-4)	6	14

The most critical finding was the incidence of clinically significant toxicity (Grade 2-4), which was substantially higher in the TOMO group (70%, 14/20) compared to the VMAT group (30%, 6/20). This difference was statistically significant (Fisher’s exact test, *p* = 0.0256). Notably, the TomoTherapy group included one case of Grade 4 ulceration, a severe adverse event not observed in any patient treated with the Synergy/VMAT technique. The Grade 4 ulceration in the TOMO group occurred within the treatment field and was managed with local wound care, with resolution occurring after radiotherapy completion.

### Skin surface dose analysis

**Figure 1:**
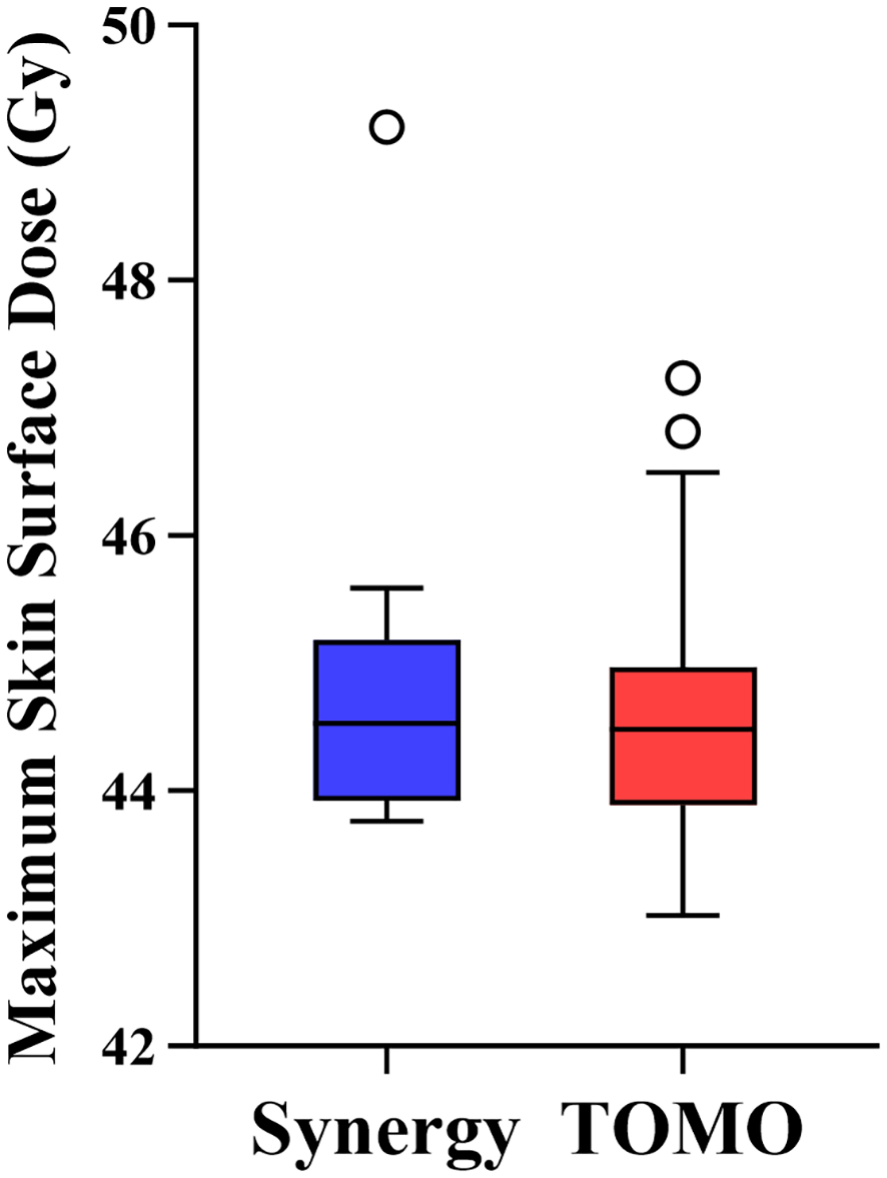
Comparison of dosimetric parameters between Synergy and TOMO groups.

Dosimetric analysis was performed to investigate the underlying cause of the differential skin toxicity. As shown in **Figure 1**, comparison of the maximum dose (Dmax) on the skin surface, derived from the Dose Surface Map (DSM) analysis, revealed no significant difference between the Synergy and TOMO groups (44.75 ± 1.20 Gy vs. 44.61 ± 1.15 Gy, p = 0.718).

### Dose distribution pattern reveals a paradox

**Figure 2:**
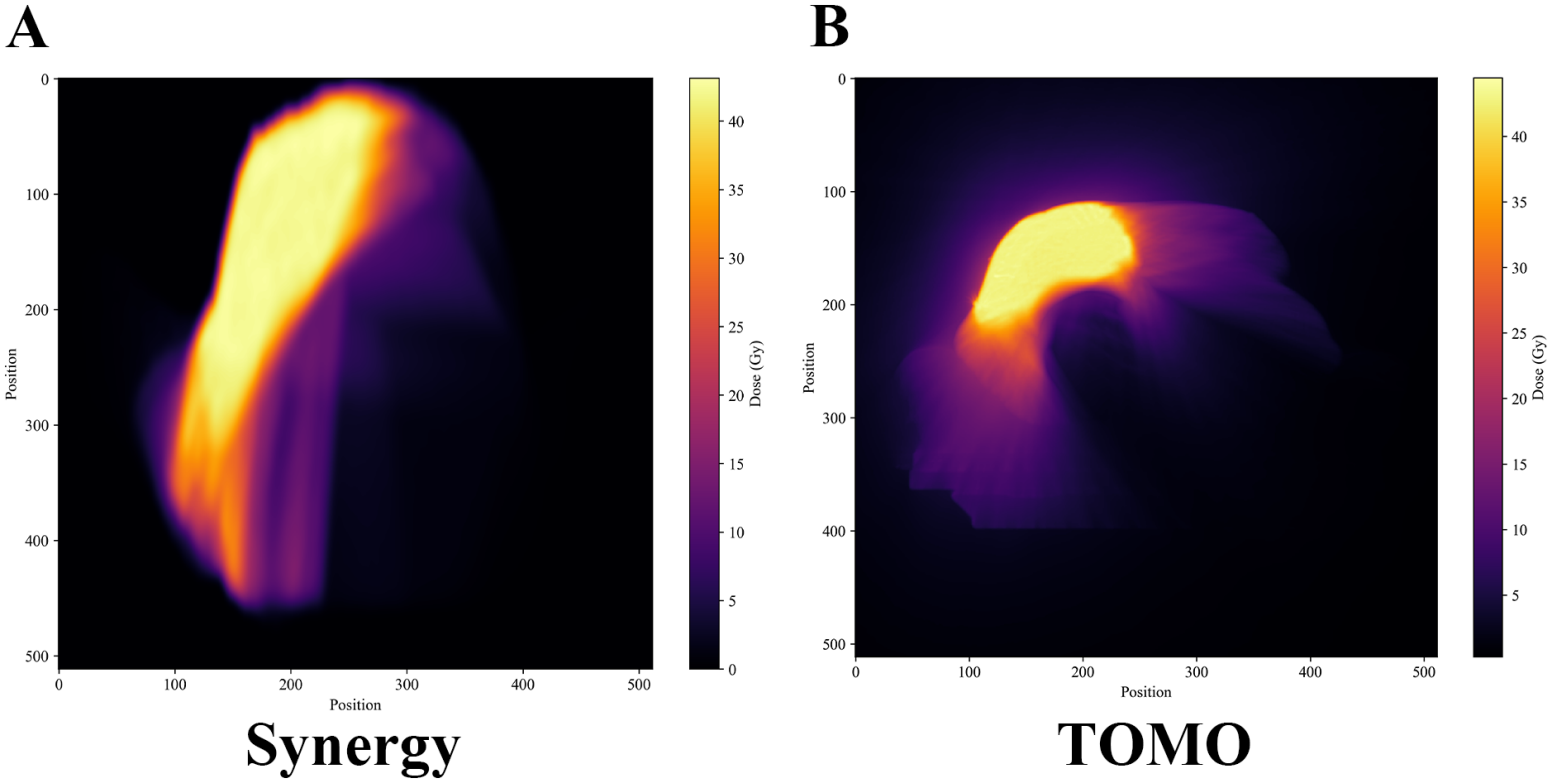
Dose Surface Maps comparison: **(A)** Synergy showing extensive high-dose distribution, **(B)** TOMO showing concentrated high-dose pattern.

However, analysis of the Dose Surface Maps from the representative patients with the most closely matched PTV volumes (as described in the Methods) revealed a counterintuitive distribution pattern (**Figure 2**). The Synergy plans demonstrated significantly more extensive high-dose regions across the skin surface, covering larger anatomical areas compared to the tightly concentrated high-dose distribution characteristic of TOMO plans.

This finding created a central paradox: the technique with more widespread high-dose regions on the skin surface (Synergy/VMAT) resulted in less severe clinical toxicity, while the technique with a more focused surface dose (TOMO) caused worse toxicity, suggesting that the spatial extent of high-dose regions on the skin surface alone cannot explain the clinical outcomes.

### P-PAST dose correlation

**Figure 3:**
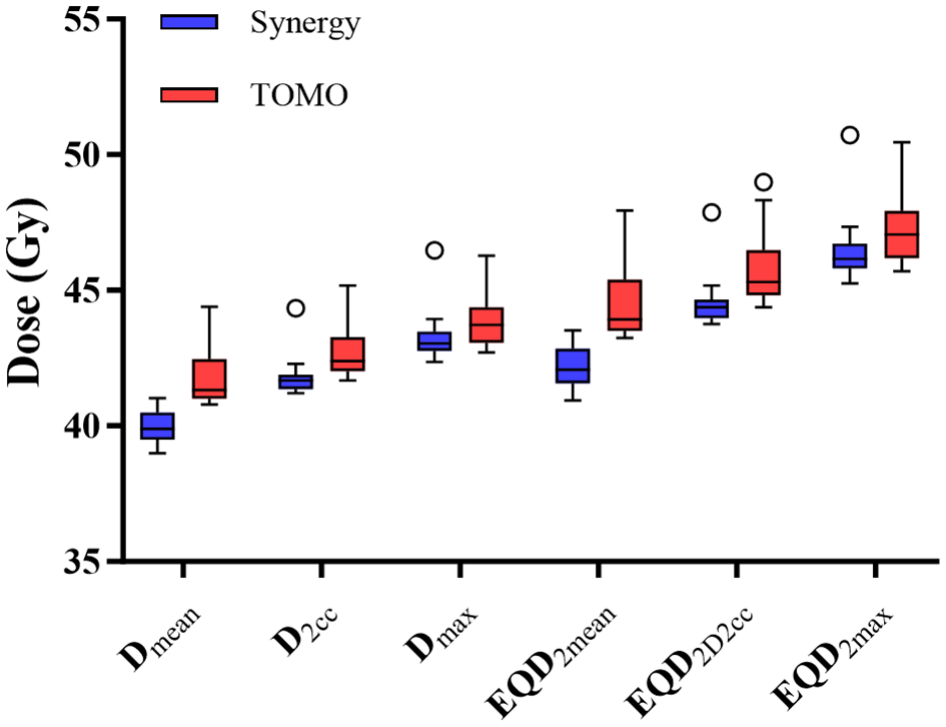
Comparison of dosimetric parameters within the PTV-projected adjacent superficial tissue (P-PAST) between Synergy and TOMO groups.

To resolve this paradox, further analysis of the PTV-Projected Adjacent Superficial Tissue (P-PAST) was performed, which provided a potential explanation for the discrepancy (**Figure 3**). The TOMO group demonstrated systematically higher dose parameters within the P-PAST region, with significantly elevated mean dose (41.74 ± 1.02 Gy vs. 39.92 ± 0.60 Gy, p< 0.001), D2cc (42.73 ± 1.00 Gy vs. 41.80 ± 0.67 Gy, p = 0.002), and maximum dose (43.86 ± 1.00 Gy vs. 43.24 ± 0.86 Gy, p = 0.046).

To assess the corresponding biological impact, equivalent doses in 2-Gy fractions (EQD_2_) were calculated. The TOMO group demonstrated significantly and consistently higher biological doses across all key parameters within the P-PAST: the mean dose EQD_2_ (44.47 ± 1.33 Gy vs. 42.12 ± 0.76 Gy, p< 0.001), the D2cc EQD_2_ (45.76 ± 1.32 Gy vs. 44.54 ± 0.88 Gy, p = 0.002), and the maximum dose EQD_2_ (47.24 ± 1.33 Gy vs. 46.43 ± 1.14 Gy, p = 0.047).

These findings provide a coherent dosimetric explanation for the clinical observations: although TOMO showed a more concentrated high-dose pattern on the skin surface, it delivered significantly higher physical and biologically effective doses to the subcutaneous P-PAST region. This elevated dose intensity in the biologically relevant dermal layer, consistently reflected across all physical and EQD_2_ parameters, correlates directly with the increased severity of acute radiation dermatitis observed in the TomoTherapy group.

## Discussion

This study elucidates the dosimetric basis for the heightened acute skin toxicity observed with TomoTherapy (TOMO) versus Volumetric Modulated Arc Therapy (VMAT). We reconciled the paradox between a confined surface dose and severe clinical toxicity by introducing a novel analysis of the PTV-projected adjacent superficial tissue (P-PAST) and its equivalent dose (EQD_2_). Our data confirm that TOMO’s unique helical delivery, while advantageous for deep-seated organ sparing, predisposes it to a synergistic dose superposition within the subcutaneous tissue (25), resulting in significant dose escalation to the biologically critical P-PAST region. This mechanistic insight, detailed below, not only explains the observed increase in dermal toxicity but also contrasts with the skin-sparing effects typically associated with other advanced techniques such as IMRT (26).

Crucially, our dosimetric data directly explain the recent large-scale clinical findings of Xia et al. (14), who reported a significantly higher incidence of severe acute skin toxicity in breast cancer patients treated with TOMO compared to IMRT. We demonstrate that the root cause lies in TOMO’s unique helical delivery geometry, which results in a pronounced dose escalation not only in physical terms but also in biological effectiveness (EQD_2_) within the dermal compartment under the target projection. This convergence of clinical and dosimetric evidence across independent studies significantly strengthens the generalizability and clinical relevance of our conclusions.

From a physical and biological standpoint, our results are consistent with the “beam angle effect” and the fundamental principles of photon dose deposition. As elucidated by Saini et al. (19), beam incidence angle critically influences skin dose. VMAT, with its relatively limited and more perpendicular beam angles relative to the breast surface, promotes dose build-up and mimics the skin-sparing advantage of the prone position. In contrast, TOMO’s helical mode employs a continuous arc of beamlets from numerous, often highly tangential angles. This geometry not only increases physical dose deposition in the build-up region but, as critically confirmed by our EQD_2_ analysis, also enhances the biological effectiveness of this dose in damaging the acutely responding skin tissues (α/β = 10 Gy), thereby amplifying the toxicity risk. The influence of such geometric factors is further underscored by the established correlation between chest wall anatomy (e.g., breast separation) and axillary/superficial dose distribution in tangential breast radiotherapy (18). The comparability of breast separation between our VMAT and TOMO groups (**Table 1**) rules out this anatomical variable as a confounder, thereby focusing the causative explanation squarely on the differential beam geometry of the two delivery systems.

Critically, the observed dosimetric differences cannot be attributed to compromised plan quality with VMAT. Our comprehensive plan quality assessment confirms that the VMAT plans in this study achieved significantly superior target conformity (higher CI, p=0.009) while maintaining equivalent dose homogeneity (comparable HI, p=0.403) compared to TOMO plans (**Table 2**). This finding is pivotal as it definitively rules out the possibility that the lower skin and P-PAST dose with VMAT resulted from under-coverage or inadequate modulation of the superficial target. Instead, it reinforces the conclusion that the pronounced subcutaneous dose escalation with TOMO is an intrinsic characteristic of its helical delivery geometry.

Furthermore, this trade-off cannot be explained by baseline patient differences, as the two groups were well-matched not only in age and PTV volume but also in key anatomical factors known to influence superficial dose, namely body mass index (BMI) and breast separation (**Table 1**). Most importantly, the observed increase in P-PAST dose with TOMO was accompanied by a quantifiable sparing benefit for deep-seated organs, directly substantiating the dosimetric trade-off. As detailed in our comparative OAR analysis (**Table 3**), TOMO plans delivered significantly lower doses to the ipsilateral lung (mean dose: 7.32 vs. 8.79 Gy, V20: 11.5% vs. 17.0%) and heart (mean dose: 1.67 vs. 2.31 Gy) compared to VMAT plans. This provides direct empirical evidence from our cohort that the helical delivery geometry, while predisposing the subcutaneous tissue to dose superposition, concurrently achieves its recognized advantage in protecting deeper structures (12, 14). Thus, the clinical dilemma—increased skin toxicity versus superior deep organ sparing—is rooted in a fundamental physical trade-off inherent to the TOMO technique. These data move the discussion from a conceptual trade-off to one that is quantitatively defined within our study population, greatly strengthening the mechanistic link between delivery geometry, dosimetry, and clinical outcome.

Our study further highlights the critical limitation of conventional two-dimensional skin dose evaluation. While our DSM results visually corroborate the findings of Zibold et al. (27)—showing a homogeneous and confined high-dose pattern on the skin surface for TOMO—this superior surface homogeneity paradoxically coincided with worse clinical toxicity. This contradiction underscores that a homogeneous surface dose is an insufficient and potentially misleading predictor of skin toxicity. Our findings therefore compel a paradigm shift: from evaluating dose on the skin surface to understanding and quantifying dose to the skin as a volumetric organ at risk. The P-PAST analysis, enriched with EQD_2_, directly addresses this need by combining the global perspective of DSM with a volumetric and biologically weighted dose assessment in the most relevant subcutaneous region.

Methodologically, the concept of analyzing a defined superficial tissue layer finds support in the work of Branchini et al. (20). Our approach enhances this methodology by specifically restricting the dose assessment to the region overlapping with the PTV (the P-PAST), thereby focusing precisely on the tissue volume at highest risk for treatment-related toxicity, rather than performing a less specific whole-body skin evaluation. The highly significant differences in both physical and EQD_2_ parameters within the P-PAST validate its utility as a robust predictor for acute skin toxicity.

The skin toxicity risk associated with TOMO appears to be an intrinsic feature stemming from its delivery system geometry. Our findings substantiate that this risk is not incidental but is fundamentally linked to the multi-angle, continuous tangential irradiation pattern, which synergistically elevates dose within the deep dermal layer. This finding aligns with the fundamental radiobiological principle that obliquely incident photon beams compromise the skin-sparing effect by reducing the dose build-up region. This principle finds direct experimental support in the work of Nilsson & Schnell, who quantitatively demonstrated that the loss of electronic equilibrium at interfaces (such as air cavities) leads to a significant increase in dose at the boundary, a physical effect directly analogous to the impact of TOMO’s highly oblique beams on the dermal compartment (28).

The primary clinical implication of our study is that for radiotherapy techniques like TOMO, plan evaluation must extend beyond skin surface Dmax and conventional organs at risk. Proactively incorporating dose-volume parameters for a structure like the P-PAST—particularly the Dmean and D2cc in EQD_2_—into the planning optimization and evaluation process is essential. This allows for a more balanced approach, enabling clinicians to pursue excellent target conformity and deep organ sparing (e.g., reduced lung dose as noted by Xia et al. (14)) while simultaneously managing the often-overlooked risk of severe acute skin toxicity, thereby achieving a more comprehensive therapeutic ratio.

This study has several limitations. Its retrospective design and moderate sample size, for which no formal *a priori* power calculation was performed, may affect the generalizability of the results. Furthermore, while key baseline and anatomical factors were well-matched between groups, a multivariable analysis to adjust for all potential confounders was not conducted. Additionally, the statistical analyses did not include adjustment for multiple comparisons, which should be considered when interpreting the secondary correlative findings. An inherent methodological consideration is the use of different dose calculation algorithms across the two platforms—Monaco employing a Monte Carlo-based algorithm for VMAT and TomoTherapy using a Collapsed Cone Convolution algorithm. Although both are advanced, clinically validated algorithms, subtle differences in their modeling of electron transport and dose deposition, particularly within the shallow build-up region (0–5 mm depth) where the P-PAST resides, could introduce a systematic uncertainty in the absolute dose comparison. Therefore, while the direction and magnitude of the observed P-PAST dose difference are robust and strongly supported by the correlated clinical toxicity outcomes, the reported absolute dose values should be interpreted with this technical context in mind. Future studies utilizing a unified dose calculation engine or phantom-based measurements in the build-up region could further refine such comparisons.

## Conclusions

In conclusion, this study resolves the clinical paradox of increased skin toxicity with TomoTherapy by identifying a pronounced dose escalation within the subcutaneous P-PAST region, which is not reflected in conventional skin surface dosimetry. The underlying mechanism is attributed to TOMO’s unique helical delivery geometry, where continuous, highly tangential beam incidence disrupts the dose build-up region and synergistically elevates both the physical and biologically effective dose to the deep dermis. These findings advocate for a paradigm shift in treatment plan evaluation, underscoring the necessity of incorporating volumetric dose assessment of the dermal compartment—specifically through parameters such as the EQD_2_ of the P-PAST—for techniques like TOMO. Such an approach is essential to balance the pursuit of superior deep tissue sparing with the mitigation of severe acute skin toxicity, ultimately optimizing the therapeutic ratio in breast cancer radiotherapy.

## Data Availability

The datasets presented in this article are not readily available because The datasets are subject to restrictions due to patient privacy and confidentiality regulations. Access is restricted to protect the sensitive clinical information of the participants. De-identified data may be made available for legitimate research purposes upon reasonable request, subject to approval by the Ethics Committee of The First Affiliated Hospital of Soochow University and the execution of a formal data use or material transfer agreement. Requests should be submitted to the corresponding author. Requests to access the datasets should be directed to Chenchen Wu, wcc2119@suda.edu.cn.
