# Correction: Beyond surface dose: subcutaneous dose escalation underlies TomoTherapy’s skin toxicity in breast radiotherapy

**DOI:** 10.3389/fonc.2026.1796339

**Published:** 2026-02-11

**Authors:** Jiao Xue, Wenjun Zhao, Chenchen Wu

**Affiliations:** 1 Department of Radiation Oncology, The First Affiliated Hospital of Soochow University, Suzhou, China; 2 State Key Laboratory of Radiation Medicine and Protection, School for Radiological and Interdisciplinary Sciences (RAD-X), Collaborative Innovation Center of Radiation Medicine of Jiangsu Higher Education Institutions, Soochow University, Suzhou, China

**Keywords:** acute skin toxicity, breast cancer radiotherapy setup, helical tomotherapy (TOMO), P-PAST, volumetric modulated arc therapy (VMAT)

There was a mistake in the captions of **Figures 1** and **3** as published. The captions for **Figures 1** and **3** were erroneously swapped. The corrected captions of **Figures 1** and **3** appear below.

**Figures 1**

“Comparison of dosimetric parameters between Synergy and TOMO groups.”

**Figures 3**

“Comparison of dosimetric parameters within the PTV-projected adjacent superficial tissue (P-PAST) between Synergy and TOMO groups.”

The original version of this article has been updated.

